# Secondhand Smoke Exposure and Inflammatory Markers in Nonsmokers in the Trucking Industry

**DOI:** 10.1289/ehp.1003199

**Published:** 2011-05-31

**Authors:** Yueh-Hsiu Mathilda Chiu, Donna Spiegelman, Douglas W. Dockery, Eric Garshick, S. Katharine Hammond, Thomas J. Smith, Jaime E. Hart, Francine Laden

**Affiliations:** 1 Exposure, Epidemiology and Risk Program, Department of Environmental Health, Harvard School of Public Health, Boston, MA, USA; 2 Channing Laboratory, Brigham and Women’s Hospital and Harvard Medical School, Boston, Massachusetts, USA; 3 Department of Epidemiology, and; 4 Department of Biostatistics, Harvard School of Public Health, Boston, Massachusetts, USA; 5 Pulmonary and Critical Care Medicine Section, VA Boston Healthcare System, Boston, Massachusetts, USA; 6 School of Public Health, University of California, Berkeley, California, USA

**Keywords:** cardiovascular disease, CRP, IL-6, inflammatory marker, nonsmoker, occupational health, plasma cotinine, secondhand smoke, sICAM-1, trucking industry

## Abstract

Background: Few studies have directly assessed the association of secondhand smoke (SHS) with cardiovascular disease–related inflammatory markers, and the findings are inconsistent.

Objectives: We assessed the association between SHS exposure and the inflammatory markers high-sensitivity C-reactive protein (hs-CRP), interleukin-6 (IL-6), and soluble intercellular adhesion molecule-1 (sICAM-1) in 199 nonsmoking U.S. trucking industry workers.

Methods: Participants provided blood samples either by mail (blood drawn at local health care provider near home) or at the work site (blood drawn by research staff on-site) and completed a health and work history questionnaire at the time of blood draw. Exposure to SHS was measured by plasma cotinine concentrations. We used multivariate regression analyses to assess the associations between levels of cotinine and inflammatory markers.

Results: The median cotinine level was 0.10 ng/mL (interquartile range, 0.04–0.23 ng/mL). The odds ratios of elevated hs-CRP (above highest CRP tertile, 1.5 mg/L) were 2.85 [95% confidence interval (CI), 1.03–7.89] for the high-cotinine group (> 0.215 ng/mL) and 2.80 (95% CI, 1.11–7.10) for the moderate-cotinine group (0.05–0.215 ng/mL), compared with the low-cotinine group (< 0.05 ng/mL), adjusting for age, sex, race, educational level, obesity, previous smoking history, job title, and medical history. Plasma cotinine levels were not associated with IL-6 or sICAM-1.

Conclusions: SHS exposure, as assessed by plasma cotinine, was positively associated with hs-CRP in this group of blue-collar workers. The strength of the association with hs-CRP depended on the cut points selected for analysis.

Secondhand smoke (SHS) is a complex mixture of chemicals generated by the burning of tobacco. SHS is associated with a number of adverse health effects, including chronic respiratory symptoms, lung cancer, and cardiovascular disease (CVD) [Barnoya and Glantz 2005; Centers for Disease Control and Prevention (CDC) 2006; Eisner and Iribarren 2007; Ho et al. 2007; Panagiotakos et al. 2002; Raupach et al. 2006]. The American Heart Association (AHA) has determined that SHS is a cause of fatal CVD (Taylor et al. 1992). Smoking prevalence has declined among adults in the United States (CDC 2000, 2005); however, among the major blue-collar occupations (e.g., construction workers, motor vehicle operators, freight handlers, mechanics), it remains higher than those of many other occupational groups (Bang and Kim 2001; Gerlach et al. 1997; Lee et al. 2007), suggesting that this is a population with high potential for SHS exposure (Wortley et al. 2002).

Several studies of systemic inflammatory markers, which have been associated with susceptibility to CVD (Blake and Ridker 2002; Saadeddin et al. 2002; Tousoulis et al. 2007), have shown positive relationships with active smoking (Melbye et al. 2007; Saito et al. 2003; Wannamethee et al. 2005). However, there is limited and inconsistent evidence of an association between SHS exposure and inflammatory markers (Clark et al. 2008; Cook et al. 2000; Nagel et al. 2009; Panagiotakos et al. 2004; Venn and Britton 2007). A study of adults in Greece (Panagiotakos et al. 2004) and another study of British elderly (Jefferis et al. 2010) without clinical evidence of CVD suggested that exposure to SHS is related to an increase in C-reactive protein (CRP) levels, whereas this association was not found among never-smoking adults in the U.S. general population (Clark et al. 2008; Venn and Britton 2007). These studies have used either self-reports or biomarkers to assess SHS exposure. Cotinine in biological fluids has been used most widely as a biomarker (Benowitz 1999; CDC 2006). It is generated from nicotine in the liver and released into the bloodstream, and thus blood levels of cotinine should closely reflect the level of nicotine from inhaled SHS (Benowitz 1999). The average half-life of cotinine is about 17 hr, and cotinine levels remain relatively stable throughout the day (Benowitz 1996). In the present study, we examined the relationship of SHS exposure measured by plasma cotinine with systemic inflammatory markers that included high-sensitivity CRP (hs-CRP) and interleukin-6 (IL-6), and with soluble intercellular adhesion molecule-1 (sICAM-1), a marker of vascular endothelial activation and inflammation, among nonsmoking workers in the U.S. trucking industry. In addition, we evaluated the relationship between self-reported SHS exposure and plasma cotinine concentrations.

## Materials and Methods

*Study participants.* Participants were from two volunteer groups of employees at unionized trucking companies in the United States in 2007. For the first group, a total of 120 nonsmoking participants were recruited on-site among active workers at two work sites (trucking terminals) located in Carlisle, Pennsylvania, and Chicago, Illinois (the on-site group). The second group of workers was recruited by mail from respondents to a health survey sent to a random sample of active and recently retired workers at three unionized companies (the mailing group). We invited these workers to provide blood samples and to complete an additional questionnaire. Of the 114 workers recruited by mail, 98 described themselves as nonsmokers. The participants represented the major job categories in the trucking industry: long-haul driver (driving between cities), pickup/delivery driver (local driving within cities), dockworker (moving freight within the trucking terminal), mechanic (repairing tractors and trailers), hostler (moving trucks in the terminal yard), and clerk (office worker) (Smith et al. 2006). The protocol was approved by the human subjects committees at the Brigham and Women’s Hospital, the Harvard School of Public Health, and the Veterans Affairs Boston Healthcare System, and each participant provided informed consent before participating.

*Blood sample collection.* For those who were recruited via mail, a blood kit containing four 10-mL liquid EDTA blood tubes, a freezer pack, and detailed instructions were sent to their households. The participants were asked to go to their local health care provider or a walk-in clinic to have approximately 30 mL of blood drawn. They then sent the blood with a coolant to our laboratory by overnight shipment. The mailing protocols were developed from the methods used in previous large epidemiologic studies that successfully collected the blood samples by mail (Pischon et al. 2003; Ridker et al. 1997). Workers recruited on-site at the trucking terminals had their blood drawn by our research team and provided blood samples before, during, or after their work shift during the sampling week. These blood samples were also sent back to the laboratory by overnight shipping every day. Upon arrival, the chilled blood samples were centrifuged in a refrigerated unit, and blood components were aliquoted in cryotubes, which were stored in liquid nitrogen freezers at less than ^–^130°C.

*SHS and health questionnaire.* Participants completed a health questionnaire, providing information regarding their exposure to SHS and previous smoking history. Detailed information about demographic characteristics and lifestyle, including age, sex, race/ethnicity, educational level, height and weight, job title and work history, time of blood draw, alcohol intake, physical activity, medical history, and medication use, was also obtained. Participants recruited by mail were asked to complete the questionnaire on the day of their blood draw and send it back to us along with the blood kit. Participants recruited on-site were asked to complete the questionnaire at the time of blood draw. Job title was categorized into four groups: drivers, terminal workers (including terminal dockworkers and hostlers), office workers, and retired.

*Assessment of SHS exposure by plasma cotinine.* SHS exposure of the workers was assessed by plasma cotinine levels. Cotinine analysis was conducted in the clinical pharmacology laboratory at San Francisco General Hospital Medical Center. Concentrations of cotinine in plasma were measured by liquid chromatography/tandem mass spectrometry (Bernert et al. 1997). The limit of quantitation (LOQ) was 0.02 ng/mL. Of 218 samples analyzed, 199 (91.3%) were > LOQ. Samples < LOQ (*n* = 19) were assigned a value of 0.014 ng/mL (the LOQ divided by the square root of 2) by the laboratory. The measurement of plasma cotinine generally reflects SHS exposure in the previous 1–2 days (Benowitz 1996).

Only workers with plasma cotinine levels ≤ 14 ng/mL were considered non smokers, because a person with a plasma cotinine level > 14 ng/mL is generally considered to be a smoker (Jarvis et al. 1987). Of 218 workers  who reported no current cigarette, cigar, or pipe smoking, 199 (91%; 94 recruited by mail and 105 on-site) had cotinine levels ≤ 14 ng/mL and were eligible for our statistical analysis. None of the participants reported taking nicotine replacement therapy.

*Assessment of inflammatory markers.* The analyses of inflammatory markers were performed at the Clinical and Epidemiologic Research Laboratory, Department of Laboratory Medicine, at Children’s Hospital (Boston, MA). CRP levels were determined by an immunoturbidimetric assay on a Hitachi 917 analyzer (Roche Diagnostics, Indianapolis, IN), using reagents and calibrators from Denka Seiken (Niigata, Japan). Concentrations of sICAM-1 and IL-6 were measured by enzyme-linked immunosorbent assays (ELISA), using the quantitative sandwich enzyme immunoassay technique (R&D Systems, Minneapolis, MN). For the three inflammatory markers analyzed, all the samples were above the laboratory thresholds of detection (0.03 mg/L for CRP, 0.094 pg/mL for IL-6, and 0.35 ng/mL for sICAM-1). The day-to-day variability of the analytic assay was 2.81% for CRP, 7.2% for IL-6, and 6.0% for sICAM-1. The Spearman’s correlation coefficient was 0.43 (*p* < 0.01) for CRP and IL-6, 0.35 (*p* < 0.01) for CRP and sICAM-1, and 0.21 (*p* < 0.01) for IL-6 and sICAM-1.

*Statistical analysis.* To evaluate the relationship between self-reported SHS exposure and plasma cotinine levels, we used linear regression analysis with adjustment for confounders. Because the residuals of this regression model were right-skewed, we used the robust variance for statistical inference (White 1980). We compared demographics, health- related characteristics, and inflammatory marker levels across three categories of plasma cotinine: < LOQ (< 0.02 ng/mL), low cotinine (0.02–0.11 ng/mL, the median value among those with quantifiable levels), and high cotinine (> 0.11 ng/mL). To determine if the levels of the inflammatory markers were different across demographic and health-related characteristics groups, we used multivariate linear regression analyses with robust variance, adjusting for potential confounders.

To assess the association between plasma cotinine and the inflammatory markers, we performed *a*) multivariate linear regression analyses, using robust variance, with inflammatory markers modeled as continuous dependent variables; and *b*) multivariate logistic regression analyses with inflammatory markers classified as dichotomous outcomes. Because there is no established cut point for defining heavy SHS exposure versus light SHS exposure, we explored several different cut-points for categorizing plasma cotinine. First, plasma cotinine was categorized into three groups (< LOQ, low, or high), as described above. In addition, we categorized cotinine using cut-points from a study of Third National Health and Nutrition Examination Survey (NHANES III) data as low (< 0.05 ng/mL, the detection limit of analytic method used in early NHANES III), medium (0.05–0.215 ng/mL, the median value among those above the detection limit in that study), or high (> 0.215 ng/mL) (Venn and Britton 2007). Tests for linear trend were performed using the median value for each category of cotinine. Finally, we also considered a cut-point of plasma cotinine derived from a receiver operating characteristic (ROC) curve (Fawcett 2006) based on self-reported SHS exposure. The ROC curve was generated by calculating sensitivity and specificity of each cotinine level in our data set, where the sensitivity represents the percentage of workers categorized as high cotinine among those who reported that they were exposed to SHS in the previous day, and the specificity represents the percentage of workers categorized as low cotinine among those who reported that they were not exposed to SHS in the previous day. The selected cut-point that maximized the specificity with a sensitivity > 70% was 0.06 ng/mL.  We considered covariates that were either biologically meaningful potential confounders or statistically significant in univariate analyses: sociodemographics (age, sex, race, educational level), job title, recruitment method, former smoking, body mass index (BMI; weight in kilograms divided by height in meters squared), and medical conditions [high blood pressure, history of heart disease, chronic obstructive pulmonary disease (COPD), chronic rhinitis, hay fever]. We also examined effect modification by age, BMI, and former smoking status by fitting interaction terms and performing stratified analyses. All *p*-values are two sided, and statistical significance was determined for an α-level of 0.05.

In logistic regression analyses, we used the median levels of each inflammatory marker as the cut-points of elevated versus nonelevated outcome variables. Additionally, the CDC and AHA suggest that the measurement of CRP in the adult population be categorized into tertiles (Pearson et al. 2003), because meta-analyses of several population-based prospective studies found that the risk of future CVD is doubled in subjects with hs-CRP in the study-specific highest tertile compared with the lowest tertile (Danesh et al. 1998, 2000; Pearson et al. 2003). Therefore, we also assessed the risk of being in the highest tertile of hs-CRP, while combining the lower two tertiles as the reference group to increase the sample size. All analyses were performed with SAS statistical software (version 9.1.3; SAS Institute Inc., Cary, NC).

## Results

*Participant characteristics.* Table 1 presents participant characteristics, as well as levels of cotinine and inflammatory markers, by SHS exposure group. Overall, the demographic characteristics of the workers were similar across the exposure groups. However, the workers recruited by mail were older (mean age, 60 years), thinner (median BMI, 27.8 kg/m^2^), less likely to report SHS exposure in the past 24 hr (36%), and more likely to report COPD (12%) and chronic rhinitis (14%), compared with those recruited on-site (mean age, 51 years; median BMI, 29.8 kg/m^2^; SHS in the past 24 hr, 68%; COPD, 5%; chronic rhinitis, 2%).

**Table 1:** Levels of plasma cotinine and inflammatory markers, and characteristics of 199 nonsmoking workers in the U.S. unionized trucking industry.

Exposure group		
Variable	All subjects (*n* = 199)	Below LOQ (*n* = 19)	Low cotinine (*n* = 90)	High cotinine (*n* = 90)
Continuous variables [median (IQR)]					
Cotinine (ng/mL)	0.10 (0.04–0.23)		N/A	0.05 (0.04–0.08)	0.26 (0.18–0.59)
CRP (mg/L)	0.93 (0.46–2.06)		0.93 (0.43–1.53)	1.02 (0.52–1.97)	0.87 (0.43–2.09)
IL-6 (pg/mL)	1.38 (1.02–2.58)		1.16 (1.04–2.05)	1.43 (1.02–3.01)	1.36 (1.02–2.26)
sICAM-1 (ng/mL)	246.1 (203.2–284.4)		288.5 (231.1–321.7)	240.5 (202.3–283.4)	243.7 (200.7–281.2)
Age (years)	57 (49–62)		58 (46–62)	57 (51–63)	55 (47–60)
BMI (kg/m^2^)*a *	28.8 (25.8–33.1)		27.4 (25.5–31.2)	29.0 (26.0–33.7)	28.9 (26.6–32.1)
Categorical variables*b *[*n *(%)]					
Male	183 (92.0)		16 (84.2)	81 (90.0)	86 (95.6)
Caucasian	183 (92.0)		16 (84.2)	85 (94.4)	82 (91.1)
Education > 12 years	71 (35.7)		9 (47.4)	30 (33.3)	32 (35.6)
SHS in previous 24 hr	105 (52.8)		9 (47.4)	34 (37.8)	62 (68.9)
Former smoker	98 (49.3)		10 (52.6)	43 (47.8)	45 (50.0)
Alcohol use in previous 24 hr*a *43 (21.6)			3 (15.8)	21 (23.3)	19 (21.1)
Job title					
Office worker	18 (9.1)		5 (26.3)	7 (7.8)	6 (6.7)
Terminal worker	58 (29.1)		6 (31.6)	21 (23.3)	31 (34.4)
Driver	97 (48.7)		5 (26.3)	44 (48.9)	48 (53.3)
Retired	26 (13.1)		3 (15.8)	18 (20.0)	5 (5.6)
Medical condition					
Heart problem/disease	24 (12.1)		3 (15.8)	10 (11.1)	11 (12.2)
High blood pressure*a*	59 (29.7)		3 (15.8)	30 (33.3)	26 (28.9)
Asthma*a*	20 (10.1)		2 (10.5)	11 (12.2)	7 (7.8)
COPD	16 (8.0)		3 (15.8)	8 (8.9)	5 (5.6)
Chronic rhinitis*a*	15 (7.5)		2 (10.5)	9 (10.0)	4 (4.4)
Hay fever*a*	43 (21.6)		3 (15.8)	26 (28.9)	14 (15.6)
Aspirin use	62 (31.2)		6 (31.6)	34 (37.8)	22 (24.4)
Exposure groups are defined by plasma cotinine levels as follows: Below LOQ, < 0.02 ng/mL; Low cotinine, 0.02–0.11 ng/mL; High cotinine, > 0.11 ng/mL. **a**Missing values: BMI (*n* = 1), alcohol use in last 24 hr (*n* = 20), high blood pressure (*n* = 3), asthma (*n* = 3), chronic rhinitis (*n* = 6), hay fever (*n* = 5). **b**Information obtained from participants’ self-reports.					

*Self-reported SHS exposure and plasma cotinine levels.* The mean ± SD and the median [interquartile range (IQR)] cotinine levels were 0.30 ± 0.60 ng/mL and 0.10 (0.04–0.23)  ng/mL for all 199 workers (Table 1). As expected, workers who reported that they were exposed to SHS in the past 24 hr had higher plasma cotinine concentrations [median (IQR), 0.18 (0.05–0.42) ng/mL] than those who reported no recent SHS exposure [0.07 (0.04–0.12) ng/mL; Wilcoxon rank-sum test *p* < 0.001]. After controlling for age, sex, race, educational level, former smoking, recruitment method, job title/work status, and the time of blood draw, self-reported SHS exposure in the past 24 hr was associated with a 0.31 ng/mL (95% CI = 0.16–0.46 ng/mL) higher cotinine level.

*Predictors of inflammatory markers.* Levels of inflammatory markers by all potential confounders are shown in Table 2. The mean ± SD and the median (IQR) hs-CRP concentrations were 1.71 ± 2.13 mg/L and 0.93 (0.46–2.06) mg/L. Only one participant had a CRP level > 10 mg/L, a level suggested as an indicator of infection (Tousoulis et al. 2007), suggesting that most of the participants did not have recent infections before the blood draw. The mean ± SD and the median (IQR) levels were 2.36 ± 3.18 pg/mL  and 1.38 (1.02–2.58) pg/mL for IL-6, and 250.9 ± 68.3 ng/mL and 246.1 (203.2–284.4) ng/mL for sICAM-1. The median levels of hs-CRP, IL-6, and sICAM-1 were 0.92 mg/L, 1.42 pg/mL, and 233.2 ng/mL, respectively, for those recruited on-site and 0.95 mg/L, 1.24 pg/mL, and 256.7 ng/mL for those recruited by mail.

**Table 2:** Levels of inflammatory markers [median (IQR)] by demographic, work-related, and health-related variables.

Variable	*n*	hs-CRP (mg/L)	IL-6 (pg/mL)	sICAM-1 (ng/mL)
Age (years)								
≤ 50		57		0.83 (0.43–1.90)		1.22 (0.84–2.05)		239.7 (202.3–282.8)
50–60		76		0.84 (0.44–1.84)		1.41 (1.08–2.42)		245.4 (198.7–297.0)
> 60		66		1.14 (0.63–2.45)		1.64 (1.05–3.39)		252.2 (214.8–283.4)
Sex								
Female		16		1.48 (0.86–3.96)		1.39 (0.99–2.48)		277.5 (237.8–328.2)
Male		183		0.91 (0.44–1.92)		1.38 (1.02–2.66)		242.4 (201.3–284.3)
Race								
Non-Caucasian		16		0.99 (0.47–3.41)		1.32 (0.83–1.83)		260.0 (190.9–297.0)
Caucasian		183		0.93 (0.46–1.96)		1.38 (1.04–2.66)		246.0 (203.7–284.3)
Education (years)								
≤ 12		128		0.98 (0.46–2.13)		1.32 (0.93–2.73)		242.0 (200.3–281.8)
> 12		71		0.83 (0.46–1.72)		1.44 (1.07–2.25)		254.2 (208.0–291.6)
BMI*a*								
Normal		27		0.71 (0.42–1.13)		1.31 (0.87–1.98)		241.6 (230.9–276.6)
Overweight		95		0.73 (0.42–1.42)		1.18 (0.88–2.05)		239.4 (199.4–279.3)
Obese		77		1.91 (0.84–3.65)		1.86 (1.16–3.60)		260.9 (208.1–301.1)
Smoking history								
Never		101		0.76 (0.42–1.69)		1.21 (0.88–2.05)		239.7 (199.0–276.6)
Former		98		1.28 (0.68–2.52)		1.73 (1.11–3.10)		255.8 (207.8–299.8)
Alcohol use in previous 24 hr								
No		136		0.98 (0.50–2.08)		1.44 (1.10–2.69)		247.7 (207.6–284.4)
Yes		43		0.73 (0.32–1.73)		1.38 (0.84–2.70)		238.9 (197.8–283.4)
Job title								
Office worker		18		1.00 (0.82–1.96)		1.31 (1.05–1.86)		282.3 (239.0–321.7)
Terminal worker		58		0.87 (0.46–1.64)		1.18 (0.88–2.10)		234.6 (203.6–268.0)
Driver		97		0.91 (0.42–1.97)		1.42 (1.03–2.99)		244.6 (198.4–283.6)
Retired		26		1.14 (0.72–2.57)		1.74 (1.18–3.52)		252.4 (214.8–306.6)
Recruitment group								
On-site		105		0.92 (0.43–2.09)		1.42 (1.03–2.66)		233.2 (197.8–273.5)
By mail		94		0.95 (0.55–1.96)		1.24 (1.01–2.57)		256.7 (217.7–299.8)
COPD								
No		183		0.86 (0.44–1.88)		1.34 (0.96–2.48)		241.6 (202.0–282.8)
Yes		16		2.49 (1.41–4.27)		1.81 (1.18–4.25)		319.3 (266.7–344.0)
Asthma								
No		179		0.91 (0.44–1.91)		1.34 (1.02–2.47)		242.8 (202.3–282.8)
Yes		20		1.89 (0.79–2.37)		1.95 (1.17–4.13)		283.9 (222.0–339.6)
Chronic rhinitis								
No		184		0.92 (0.45–1.95)		1.32 (0.98–2.48)		242.0 (203.1–284.0)
Yes		15		1.86 (0.71–2.17)		2.15 (1.65–2.91)		274.3 (242.8–316.9)
High blood pressure								
No		140		0.82 (0.44–1.88)		1.22 (0.88–2.07)		242.6 (202.6–284.0)
Yes		59		1.38 (0.71–2.68)		2.05 (1.16–3.34)		256.6 (217.2–314.1)
Heart problem/disease								
No		175		0.93 (0.46–1.96)		1.32 (1.02–2.48)		241.6 (202.3–281.2)
Yes		24		1.18 (0.54–2.41)		2.07 (1.08–4.62)		287.0 (210.6–358.9)
Hay fever								
No		156		0.90 (0.45–1.90)		1.36 (1.03–2.65)		246.8 (205.1–289.9)
Yes		43		1.22 (0.50–2.16)		1.40 (0.88–2.48)		231.2 (202.0–282.8)
Aspirin use								
No		137		0.93 (0.46–1.91)		1.34 (1.04–2.48)		242.4 (200.7–282.8)
Yes		62		0.89 (0.46–2.37)		1.41 (0.92–2.91)		254.2 (208.0–290.2)
**a**Normal, < 25 kg/m^2^; overweight, 25–29.9 kg/m^2^; obese, ≥ 30 kg/m^2^.								

The results from multivariate linear regression analysis also revealed other important significant predictors of inflammatory markers, including obesity (BMI ≥ 30 kg/m^2^, for all three markers), former smoking (for hs-CRP), COPD (for hs-CRP and sICAM-1), ≥ 60 years of age (for IL-6), and Caucasian (for IL-6) (data not shown). The multivariate-adjusted odds ratio (OR) of high CRP (highest tertile) comparing participants who reported that they were exposed to SHS with those who reported no SHS exposure in the past 24 hr was positive but not significant [OR = 1.10 (95% CI, 0.51–2.35)].

*Plasma cotinine and inflammatory markers.* In the multivariate linear regression analyses assessing the relationship between continuous levels of plasma cotinine and each inflammatory marker, we found no statistically significant evidence of a positive linear association after controlling for age, sex, race, educational level, job title/work status, recruitment method, former smoking, obesity, high blood pressure, history of heart disease, COPD, chronic rhinitis, and hay fever (data not shown). In the multivariate logistic regression adjusted for the same covariates listed above, we found a positive but not statistically significant association between SHS exposure, as defined by continuous cotinine levels, and elevated (highest tertile vs. others) hs-CRP (OR = 1.15; 95% CI, 0.95–1.39, associated with a doubling in cotinine concentration). Table 3 presents the results from multivariate-adjusted logistic regression models using the different exposure cut-points, where we also observed a suggestive independent positive relationship between categorical cotinine and hs-CRP. However, the results varied based on the cut-points of plasma cotinine used. In addition, when we defined “elevated hs-CRP” as being in the highest tertile, we observed a stronger and significant positive association with cotinine levels > 0.05 ng/mL. Overall, we found no evidence of a significant association of plasma cotinine with IL-6 or sICAM-1, including when we explored other cut-points such as the highest tertile.

**Table 3:** Cotinine levels in relation to elevated inflammatory markers in nonsmoking U.S. trucking industry workers.

Inflammatory marker/cotinine level	Multivariate adjusted*a *			
	*n*	OR (95% CI)		*p*-Value for trend
hs-CRP (> median vs. ≤ median)*b *							
Truck industry							
Below LOQ, < 0.02 ng/mL		19		Reference			0.84
Low, 0.02–0.11 ng/mL		90		1.05	(0.32–3.48)		
High, > 0.11 ng/mL		90		1.12	(0.33–3.84)		
NHANES III							
Low, < 0.05 ng/mL		65		Reference			0.80
Moderate, 0.05–0.215 ng/mL		79		1.78	(0.77–4.09)		
High, > 0.215 ng/mL		55		1.43	(0.57–3.60)		
ROC curve							
Low, ≤ 0.06 ng/mL		73		Reference			0.08
High, > 0.06 ng/mL		126		1.99	(0.92–4.30)		
hs-CRP (highest tertile vs. others)*c *							
Truck industry							
Below LOQ, < 0.02 ng/mL		19		Reference			0.21
Low, 0.02–0.11 ng/mL		90		1.18	(0.31–4.59)		
High, > 0.11 ng/mL		90		1.88	(0.48–7.35)		
NHANES III							
Low, < 0.05 ng/mL		65		Reference			0.17
Moderate, 0.05–0.215 ng/mL		79		2.80	(1.11–7.10)		
High, > 0.215 ng/mL		55		2.85	(1.03–7.89)		
ROC curve							
Low, ≤ 0.06 ng/mL		73		Reference			0.02
High, > 0.06 ng/mL		126		2.72	(1.16–6.37)		
IL-6 (> median vs. ≤ median)*d *							
Truck industry							
Below LOQ, < 0.02 ng/mL		19		Reference			0.70
Low, 0.02–0.11 ng/mL		90		1.12	(0.35–3.65)		
High, > 0.11 ng/mL		90		0.94	(0.28–3.14)		
NHANES III							
Low, < 0.05 ng/mL		65		Reference			0.94
Moderate, 0.05–0.215 ng/mL		79		1.60	(0.69–3.71)		
High, > 0.215 ng/mL		55		1.21	(0.49–2.99)		
ROC curve							
Low, ≤ 0.06 ng/mL		73		Reference			0.18
High, > 0.06 ng/mL		126		1.70	(0.78–3.70)		
sICAM-1 (> median vs. ≤ median)*e *							
Truck industry							
Below LOQ, < 0.02 ng/mL		19		Reference			0.61
Low, 0.02–0.11 ng/mL		90		0.48	(0.15–1.53)		
High, > 0.11 ng/mL		90		0.70	(0.21–2.26)		
NHANES III							
Low, < 0.05 ng/mL		65		Reference			0.15
Moderate, 0.05–0.215 ng/mL		79		1.32	(0.60–2.91)		
High, > 0.215 ng/mL		55		0.66	(0.28–1.55)		
ROC curve							
Low, ≤ 0.06 ng/mL		73		Reference			0.80
High, > 0.06 ng/mL		126		1.10	(0.54–2.22)		
Exposure cut-points for NHANES III are from Venn and Britton (2007); those for the ROC curve were calculated following the method of Fawcett (2006). **a**Logistic regression model adjusted for age, sex, race, educational level, job title, recruitment method, former smoking, obesity, high blood pressure, history of heart disease, COPD, chronic rhinitis, and hay fever. **b**hs-CRP median, 0.93 mg/L. **c**hs-CRP highest tertile, > 1.5 mg/L. **d**IL-6 median, 1.38 pg/mL. **e**sICAM-1 median, 246.1 ng/mL.							

We found some evidence of effect modification by obesity from stratified analyses, although the interaction between obesity and cotinine was not statistically significant (*p* for interaction = 0.49). The OR (95% CI) of elevated CRP (highest tertile) comparing the high-cotinine (> 0.06 ng/mL) group with the low-cotinine group was much higher in obese workers [*n* = 77; OR = 6.65 (1.55–28.6)] than in nonobese workers [*n* = 122; OR = 1.51 (0.43–5.24)]. We found no statistically significant evidence of effect modification by age, sex, and former smoking (data not shown). We also performed an analysis restricted to the workers recruited on-site (*n* = 105), because these workers were all currently working. In addition, detailed information on other potential confounders, including physical activity and use of cholesterol control medicine, was available only for this group. The results were similar to the multivariate logistic regression analyses performed for all participants, and additional inclusion of these potential confounders did not alter the results. Similarly, the conclusions from the models restricted to males (*n* = 183) did not differ from the final model including both males and females (data not shown). Furthermore, because recent infections or illness might increase the CRP level (Tousoulis et al. 2007), we performed an analysis restricted to participants who did not report a recent illness (*n* = 182), and an analysis excluding one participant whose CRP was > 10 mg/L. The results were consistent with analyses for the population as a whole.

## Discussion

In this population of nonsmoking blue-collar trucking industry workers, we observed evidence of an association between plasma cotinine levels and elevated hs-CRP, but not IL-6 or sICAM-1. Self-reported SHS exposure in the past 24 hr was significantly positively associated with increased level of plasma cotinine, indicating that self-reported SHS exposure was reliable in this population.

A few previous studies have examined the association between SHS exposure and CRP, with inconsistent results. In studies of NHANES III, no significant association was found between CRP and serum cotinine levels in never-smokers in the U.S. general population (Venn and Britton 2007) or nonsmoking adult workers without home SHS exposure (Clark et al. 2008). On the other hand, in an analysis of Greek adults who had never smoked (*n* = 995), Panagiotakos et al. (2004) reported a 0.8 mg/L (*p* = 0.03) increase of CRP for those regularly exposed to SHS (> 3 days/week for at least 30 min/day), compared with those who were not exposed. In another study, Jefferis et al. (2010) investigated about 5,000 nonsmoking British elderly and observed a significant association between serum cotinine and CRP in a linear regression model adjusted for age, sex, region of residence, health behaviors, social class, and BMI (β = 0.03; 95% CI, 0.01–0.05; difference of natural log–transformed CRP associated with a doubling in cotinine concentration). The results from the present study suggest that higher plasma cotinine levels (> 0.05 ng/mL) may be associated with elevated CRP (> 1.5 mg/L). The analytic method for CRP used in our study is a high-sensitivity immunoassay with a detection limit of 0.03 mg/L, compared with 3 mg/L (Venn and Britton 2007) and 0.1 mg/L (Clark et al. 2008) in the NHANES III studies.

To our knowledge, the association between SHS exposure and IL-6 was investigated previously only in a study of British elderly (Jefferis et al. 2010). In multivariate-adjusted linear regression, that study showed a marginally significant positive association between log-transformed IL-6 and cotinine (β = 0.01; 95% CI, 0.00–0.02; associated with a doubling in cotinine concentration), whereas our study did not show this association. No previous studies have estimated the effect of SHS exposure on sICAM-1, and we did not observe an association in the present study. Other markers of inflammation, such as homocysteine and fibrinogen, have been associated with serum cotinine levels (Clark et al. 2008; Jefferis et al. 2010; Panagiotakos et al. 2004; Venn and Britton 2007). It is possible that the  biomarkers of inflammation that we examined are less sensitive to low level SHS exposure than are homocysteine and fibrinogen.

Our findings that plasma cotinine may be associated with CRP but not with the other two inflammatory markers may partially be explained by the stability of each measured biomarker. CRP has a half-life of about 19 hr (Koenig et al. 2003), similar to that of plasma cotinine (Benowitz 1996), and may require an induction time of 1–2 days after an acute inflammatory stimulus (Rückerl et al. 2007). Therefore, an elevation in CRP may reflect SHS exposure about 2–3 days before, which is close to the suggested exposure period corresponding to measured plasma cotinine (Benowitz 1999). On the other hand, IL-6 has a shorter half-life (2–6 hr) (Riches et al. 1992) and likely reflects a more immediate response. There is limited information to date on sICAM-1 to make an inference of the exposure–response time line. It is possible that plasma cotinine may not be an appropriate measurement to determine if an association exists between SHS exposure and IL-6 or sICAM-1.

One potential problem in studies examining health effects among nonsmokers is that smokers might be misclassified as nonsmokers and included in the study population. We used 14 ng/mL as the cut-point of plasma cotinine levels for distinguishing smokers from nonsmokers, as suggested previously by Jarvis et al. (1987). Recently, Benowitz et al. (2009) suggested that this definition might overestimate the number of nonsmokers and that the cut-point should be lowered and be specific to sex and race/ethnicity. They proposed an optimal cut-point of 6.79 ng/mL for adult non-Hispanic white males, most of our study population. In the present study, after excluding workers with cotinine levels > 14 ng/mL, the maximum level of plasma cotinine measured in our study population was 4.44 ng/mL, which is well below this optimal cut-point. Thus, those defined as nonsmokers in our study would still have been identified as nonsmokers even with this lower cut-point.

To our knowledge, there is no well-established cut-point of cotinine for high/heavy SHS exposure versus low/light SHS exposure. Previous studies have used median levels or levels of detection as cutoffs (Clark et al. 2008; Venn and Britton 2007), which are specific to the study population and the laboratory analytic methods. In our study, we explored the association between plasma cotinine and inflammatory markers using several different cotinine cut-points, including the LOQ (0.02 ng/mL), the median value, and an optimal cutoff chosen from a ROC curve based on data from our population, and cut-points used in previous studies of NHANES III data (Clark et al. 2008; Venn and Britton 2007). Although the ORs of elevated hs-CRP were positive when 0.02 ng/mL was used as the cut-point for the low cotinine group, this effect estimate was not statistically significant, probably because this cut-point is too low and the group size is too small. The ROC cut-points and the NHANES cut-points seemed to produce consistent results, possibly at least in part because of the similar cut-points for the reference exposure group. Although exploring different cutoffs could increase the possibility of observing an association due to chance, we found a consistent pattern of a positive association between cotinine and CRP levels across all the analyses regardless of the cutoffs.

Previous studies have derived some clinical cut-points for CRP. It has been suggested that a person is at low risk of CVD if CRP is < 1 mg/L, average risk if CRP is 1–3 mg/L, and high risk if CRP is > 3 mg/L, and these cut-points approximately correspond to tertiles of CRP levels in the general population of U.S. adults (Pearson et al. 2003; Tousoulis et al. 2007). In addition, a meta-analysis of a list of prospective studies showed that subjects in the highest tertile (study-specific cut-points) of hs-CRP in general had twice the relative risk of major coronary events than those in the lowest tertile (Danesh et al. 1998, 2000). Thus, the CDC/AHA suggested that the measurement of CRP in the adult population be stratified into three tertiles (Pearson et al. 2003; Tousoulis et al. 2007). However, there are no well-established cut-points for sICAM-1 or IL-6. In the present study, the median of hs-CRP was 0.93 mg/L, which was close to 1 mg/L, the clinical cut-point of average risk of CVD versus low risk of CVD. To be consistent, we also used the median as a cut-point for sICAM-1 and IL-6 in our analyses. In addition, we explored different clinical cut-points of hs-CRP suggested by the studies mentioned above. We found a stronger association between SHS exposure and hs-CRP when we compared the highest tertile of hs-CRP with the lower two tertiles.

Our study has several potential limitations. The measurement of plasma cotinine generally reflects the SHS exposure in the previous 1–2 days and might not represent more acute or more chronic exposures if the exposure is largely variable. Previous studies have suggested that IL-6 levels have a large daily variability (Tousoulis et al. 2007), whereas sICAM-1 and CRP do not (Mosevold and Bruserud 2002; Osmancik et al. 2004; Tousoulis et al. 2007). It is possible that we did not completely account for the circadian variation, but the results of our analyses were unchanged when the time of blood draw (i.e., morning, afternoon, evening, overnight) was included in our regression models (data not shown). Our data came from two recruitment sources. Members of the mailing group had their blood samples collected independently using protocols developed for large epidemiologic studies (e.g., the Nurses’ Health Study) and returned them by express mail (Pischon et al. 2003; Ridker et al. 1997). The blood from the on-site group was collected by our study team and similarly express mailed to our laboratory for processing. Despite these collection differences, the distributions of inflammatory markers were similar between the two recruitment groups (Table 2). We also controlled for participant characteristics and the recruitment method in all of the multivariate regression analyses. In addition, the results from the sensitivity analyses restricted to the on-site group were similar to the findings of the analyses performed for all participants.

The range of plasma cotinine and some covariates (e.g., BMI, age) we observed in this study was relatively narrow compared with the NHANES III studies, so we might have missed the association between SHS exposure and inflammatory markers because of nondifferential misclassification. Nevertheless, we used high-sensitivity laboratory analytic methods for both plasma cotinine and inflammatory markers, minimizing the chance of misclassification for both exposure and outcome. The sample size of our study was small, leading to the possibility of insufficient power to detect some potentially significant differences. Moreover, other occupational exposures, such as diesel exhaust, may confound or modify the association of SHS exposure with the inflammatory markers. However, we adjusted for job title, a reasonable indicator of occupational exposure to air pollutants in these trucking companies (Smith et al. 2006). Measurements of some conventional biomarkers related to coronary heart disease, such as lipid levels (low-density lipoprotein, high-density  lipoprotein, and triglycerides), were not available for our study. However, these are unlikely to confound the association between SHS and inflammatory markers because it is unlikely that these factors would be an antecedent or a proxy marker of an antecedent of SHS. Although information on a history of heart problems and high blood pressure was based on self-report, as opposed to medical records or direct measurements, workers in these trucking companies are required to pass a physical examination conducted every 1–2 years in order to continue working, and health conditions such as high blood pressure and any heart diseases have to be treated. Some retired workers were recruited by mail, but they were all recently retired (within 5 years of the study period). Finally, socioeconomic characteristics might cause residual confounding. However, our study population is a group of blue-collar workers with fairly homogeneous backgrounds, and we adjusted for years of education. Thus, social factors are unlikely to affect the results we observed in this study.

## Conclusion

We observed a positive relationship between SHS exposure, as measured by cotinine levels in plasma, and hs-CRP among nonsmoking workers in the trucking industry, although the strength of this association depended on the cut-points selected for analysis. We found no association of plasma cotinine with sICAM-1 or IL-6. These results may reflect the relative half-lives of the measured biomarkers and may depend on the methods used to define cutoffs of exposures and health effects.
